# New York

**Published:** 1895-08

**Authors:** 


### LEGISLATION.

### New York.

While New York State has been dilatory in securing veterinary legislation, and has, considering the importance of its live-stock interests, been far behind many other States, she has at last accomplished some very important results. The passage of an act relieving veterinarians from jury duty, and the defeat of a proposed act extending the time of registration for non-graduates, which were obtained last winter, have both been already reported to our readers.

The far more important act establishing a Board of Veterinary Examiners in connection with the Regents of the University of State of New York, was passed the end of the session, signed by the Governor, and became a law July 1, 1895.

In compliance with this act the New York State Veterinary Medical Society called a meeting in New York City on June 11, 1895, to elect the candidates from whom the Regents selected the Board of Examiners. The Society selected the names of Drs. R. S. Huidekoper, N. P. Hinkley, James Law, C. D. Morris, W. H. Kelly, John Wende, W. L. Baker, J. A. Bell, Arthur O'Shea, and H. S. Hanson. From these the Regents appointed the first five to be the first Examining Board.

A series of circulars have been issued for instruction as to the interpretation of the new law and for guidance for applicants for examination under its provisions, from which we give the following extracts. It is to be understood at the outset that all veterinarians *legally* registered in the county clerk's office prior to July 1, 1895, according to the laws of 1886 and 1893, are entitled to continue the practice of veterinary medicine; that any persons *illegally* registered can still be prosecuted; and that in future (after July 1, 1895) no person shall commence the practice of veterinary medicine in New York State without complying with the provisions of the new law.

FORM 1. *Application for License to Practise Veterinary Medicine.*

I hereby apply for license to practise veterinary medicine in the State of New York, and inclose the following proofs and fee as required by Article 10 of the Public Health Law as amended in 1895: 1, required evidence as to age; 2, certificates of moral character; 3, required evidence of preliminary education; 4, required evidence of veterinary education; 5, certified check, post-office order, or express money order for $10. (Cancel words not applying.) Make checks, drafts, etc., payable to the University of the State of New York. (Signature of applicant. . . . . P. O. address . . . .)

*Evidence of Veterinary Medical Education Required from Candidate for License to Practise Veterinary Medicine in the State of New York.*

1, Full name; 2, date of birth; 3, give the date and source of each veterinary credential (diploma, license, or degree) which you hold; 4, what diploma or license, if any, conferred on you full right to practise veterinary medicine? 5, how many years have you studied veterinary medicine?

FORM 2. *Certificates of Good Moral Character.* (*Signed by not less than two veterinarians in good standing.*)

This certifies that I have been personally acquainted with Dr. . . . for . . . years; that I believe him to be of good moral character and I hereby recommend him to the Regents of the University as entirely worthy to be licensed to practise veterinary medicine in the State of New York, pursuant to law.

FORM 3. *Certificate of Preliminary and Veterinary Education.*

[Either this certificate, properly made out, signed, and sealed by the president or secretary of the veterinary school, or both preliminary and professional original credentials must accompany application for admission to a licentiate examination.]

It is hereby certified that . . . holding veterinary student certificate No. . . . on . . . 189 , received from . . . a diploma conferring on him a degree as veterinarian, and that he studied veterinary medicine at least three full years, including three satisfactory courses of lectures in different years, as follows:

| Months. | Year. | Name of Institution. |
|---|---|---|
| to | 18 | |
| to | 18 | |
| to | 18 | |

In witness whereof, I hereunto set my . . . hand and the seal of . . . this . . . day of . . .

*Requirements for License to Practise Veterinary Medicine in New York State.*

All requirements for admission should be completed at least one week before examinations. They are as follows:

1. Evidence that applicant is more than twenty-one years of age (Form 1).

2. Certificates of moral character from not less than two veterinarians in good standing (Form 2).

3. Evidence that applicant has the general education required, preliminary to receiving a degree in veterinary medicine in this State (Veterinary student certificate. See enclosed examination handbook).

*First exemption:* "Students who had matriculated in a veterinary medical school before October 1, 1895, shall be exempt from this preliminary education requirement, provided the degree be conferred before July 1, 1898." (Form for exemption certificate furnished on application.)

4. Evidence that applicant "has studied veterinary medicine not less than three full years, including three satisfactory courses, in three different academic years, in a veterinary medical school registered as maintaining at the time a satisfactory standard" (Form 1).

*Second exemption:* "The regents may in their discretion accept, as the equivalent for any part of the 3d and 4th requirement, evidence of five or more years' reputable practise of veterinary medicine, provided that such substitution be specified in the license.

5. Evidence that applicant "has received a degree as veterinarian from some registered veterinary medical school" (Form 3 or original credentials).

6. The candidate must pass examinations in comparative anatomy, physiology and hygiene, chemistry, veterinary surgery, obstetrics, pathology and diagnosis, and therapeutics, practise, and materia medica.

*Third exemption:* "Applicants examined and licensed before July 1, 1897, by other State examining boards registered by the regents as maintaining standards not lower than those provided by this article, and applicants who matriculate in a New York State veterinary medical school before July 1, 1896, and who receive the veterinary degree from a registered veterinary medical school before July 1, 1897, may without further examination, on payment of $10 to the regents and on submitting such evidence as they may require, receive from them an indorsement of their licenses or diplomas conferring all rights and privileges of a Regent's license issued after examination."

7. A fee of $10, payable in advance.

*Veterinary Examinations.*

Examinations for license to practise veterinary medicine in this State will be held September 24–27, 1895; January 28–31, 1896; April 7–10, 1896; May 19–22, 1896; June 16–19, 1896. At the following places: New York, Albany, Syracuse, Buffalo. Each candidate is notified as to exact place.

*Morning*, 9.15–12.15. *Tuesday*, comparative anatomy; *Wednesday*, chemistry; *Thursday*, obstetrics; *Friday*, therapeutics, practice, and materia medica.

*Afternoon*, 1.15–4.15. *Tuesday*, physiology and hygiene; *Wednesday*, veterinary surgery; *Thursday*, pathology and diagnosis.

The Board of Veterinary Examiners are further empowered to receive, charge, and hear evidence, and upon proper ground to recommend to the Regents the revoking of licenses to practise, which the Regents may execute.

It will be seen that this law allows ample time for graduates of two-year schools, and for those who have graduated without the requisite preliminary education to take advantage of the exceptions for the next year, but demands rigidly that after 1898 all veterinarians to be licensed shall have the requisite education. As the execution of this law is placed in the hands of the State Society and of the powerful Regents, and as ample provision is made for punishment of any infringement of its paragraphs, by both fine and imprisonment, it is believed that it will be rigidly enforced.

